# Implementing Federalism in the Health System of Nepal: Opportunities and Challenges

**DOI:** 10.15171/ijhpm.2018.121

**Published:** 2018-12-22

**Authors:** Rajshree Thapa, Kiran Bam, Pravin Tiwari, Tirtha Kumar Sinha, Sagar Dahal

**Affiliations:** ^1^Deutsche Gesellschaft für Internationale Zusammenarbeit (GIZ) GmbH, Kathmandu, Nepal.; ^2^FHI 360 Nepal, LINKAGES Nepal Project, Kathmandu, Nepal.; ^3^Province Health Directorate, Ministry of Social Development, Dhankuta, Nepal.

**Keywords:** Challenges and Opportunities, Decentralization, Federalism, Health Reform, Health Sector, Nepal

## Abstract

Nepal moved from unitary system with a three-level federal system of government. As federalism accelerates, the national health system can also speed up its own decentralization process, reduce disparities in access, and improve health outcomes. The turn towards federalism creates several potential opportunities for the national healthcare system. This is because decision making has been devolved to the federal, provincial and local governments, and so they can make decisions that are more representative of their localised health needs. The major challenge during the transition phase is to ensure that there are uninterrupted supplies of medical commodities and services. This requires scaling up the ability of local bodies to manage drug procurement and general logistics and adequate human resource in local healthcare centres. This article documents the efforts made so far in context of health sector federalization and synthesizes the progress and challenges to date and potential ways forward. This paper is written at a time while it is critical to review the federalism initiatives and develop way forward. As Nepal progress towards the federalized health system, we propose that the challenges inherent with the transition are critically analysed and mitigated while unfolding the potential of federal health system.

## Background

With the promulgation of its constitution in 2015, Nepal replaced a unitary government with a federal system of government.^1^ This process has made Nepal a federal democratic republic governed with three levels of government: a federal level, seven provinces and 753 local government.^2^ It also led to local elections in late 2017 for the first time in two decades, a landmark achievement signalling that federalism is being implemented.^2^ With the ongoing restructuring of the health system delivery, this article aims to synthesize the progress and challenges to date and potential ways forward based on the perspective of the federal government. The progress of federalization in Nepal is also considered in light of the experiences of other countries that have implemented federalism, and these remarks may be pertinent to progressively guide the management of the health sector federalization.

## Current Situation in Nepal

During the last few decades, Nepal Government has made significant progress towards reducing maternal, under-five and infant mortality rates. During the same period, Nepal was able to halt and reverse the trends of tuberculosis, HIV and malaria with elimination of polio, maternal and neonatal tetanus and leprosy. For instance in 2016, infant and child mortality reduced from 46 to 32 and from 54 to 39 per 1000 live births respectively within the last five years.^3,4^ Despite this progress, equitable access to healthcare is still a major challenge. The rise of non-communicable diseases, including mental health, natural disaster induced health problems and an increasing number of deaths and injuries due to road accidents are an increasing challenge that the health sector needs to respond to effectively and efficiently.^5^

Nepal aims to accelerate universal health coverage (UHC). The National Health Policy of Nepal (2014)^6^ aims to improve access to quality and equitable health services and provide basic healthcare services (BHCS) free of charge while non-BHCS will be covered through the social health insurance. This aim is reflected in strategic papers, including the Nepal Health Sector Strategy 2015-2020^5^ which outlines four strategic directions: health system reform, equitable access, improved quality of services, and multi-sectoral approaches. The high out of pocket expenditure is a major barrier to health service use for many Nepali people. Approximately 10.7% of the population have experienced catastrophic financial exposures, with 1.67% of the population falling below the poverty line because of health expenditures.^7^

The health sector reform has been identified as a core strategy of two consecutive Nepal Health Sector Strategy 2004 and 2010 to respond to the changing health need of increasing population, urbanization and emerging triple burden of diseases.^8,9^ Despite continued effort only incremental reform initiatives were dominant. In 2017, there were the elections of local bodies with a constitutionally allotted executive, judicial and legislative powers. The constitutional provisions are elaborated in the Functional Assessment and Analysis (FAA)^10^ and the local Government Operation Act 2017.^11^

Shifting executive power to local and provincial level is an approach documented to have positive effects on local resource usage through participatory bottom-up planning, increased accountability and reduced bureaucracy in decision making in other countries,^12^ however, the prospective gains are contextual and not guaranteed. Similar reforms in Ghana, Zambia, Uganda, the Philippines, and Pakistan have at times exacerbated inequities, weakened local commitment to priority health issues, and interfered with service delivery by disrupting referral chains.^13-15^ Such outcomes threaten accessibility, including in controversial specialties susceptible to local pressures (eg, family planning) and those that require a well-functioning health system (eg, emergency care).^16^ Capacity-building and accountability are known to be essential to decentralized health system performance.^17,18^ The organization of health system among countries is diverse, which means there is no such one-size-fits-all organization of health service in a federal context^19^ and health system performance ranking of federalized countries is quite varied.^20^ In this context, Nepal need to develop its own tailor-made model of federalism.

In September 2015, the Ministry of Health and Population (MoHP) formed a high-level committee and Technical Working Group for functional analysis of each level of the government, health system restructuring, and transition management. It completed the functional analysis and also outlined the broad structure framework for three tiers of government. However, it could not dig out over transition management. The ministry also formed the Federalism Implementation Unit^21^ to facilitate health sector federalization, address the issues while transitioning and largely it continued the work of Technical Working Group. The 2018-2019 fiscal year budget provides promising scope for improving the health system. Of the total NPR 1.31 trillion national budget, 56.42 billion (4.29 %) has been allocated to the Ministry of Health and Population. Similarly, NPR 113 billion (8.6%) and NPR 195 billion (14.8%) has been transferred to the province and local government respectively from the federal budget.^22,23^

## Current Challenges

A key concern associated with implementing federalism is the de-prioritization of the social sector, including health, over other needs. This was clear in the case of Uganda, where the local government health planners allocated declining proportions of budgets to public services.^24^ In case of Nepal, to overcome the issue of de-prioritisation of health sector, a very positive practice was adopted in the first and second year of the budget allocation, as federal government channelled the health sector budget under conditional grant which allowed for uninterrupted delivery of the priority health interventions.^22^ The constitution envisions four types of resource allocation frameworks^2^; equalization, conditional, special and complementary grants. To this date, only two grants: conditional and equalization disbursed through federal government are in operation, however the resource generated from the local government is also being used at the local level. The issue of health sector prioritization will continue to be a concern across all three levels of government and different grants including the special and complementary grants.

Of special concern in federalism are spill-over effects and lack of clarity in the delineation of authority between jurisdictions in the different layers of government.^25^ This is an acute concern for Nepal, with many local governments (753), with a diverse range of economic potentials, holding executive, legislature, and financial procedural power. The health system in high-performing federal countries is usually organized through federal legislation^26^; therefore, it is critical to accelerate the pace of developing federal legislative framework in Nepal.

Another major challenge is human resource management. Resistance of civil servants to change in the power structure has always been a challenge in health system decentralization.^27^ The health service delivery structure in Nepal was developed at the time when the country’s population was only 10 million. The present health structures and human resource base of 35 thousand people is not sufficient to provide adequate health service delivery in context of changing burden of disease, growing advancement in healthcare technologies and increasing population.^28-30^ To cope with this, increasing the desired human resource through recruiting on a temporary base and relying on the private sector was the dominant approach in the last decade.

Further, the number of sanctioned positions at the federal level has been downsized: MoHP reduced to 106 from 111 and Department of Health Services (DoHS) reduced to 121 from 196^31^ before the structures at the province level is fully functional and responsive. Further, the health structure at the local level is not endorsed yet. The previous health function delivered through district health offices are now to be delivered through new structure at sub national level. The deputed health personnel at local level (eg, paramedics at service outlets) are primarily trained to offer health services and therefore, lack skills on management and procurement at large. This requires extensive capacity building pertaining to planning, monitoring, evaluation and overall management of the health service delivery. In line with the aspiration of the constitution and the functional analysis, a tailor made appropriate health service delivery structures and the right staff mix is critical.

The constitution assigns the management of BHCS to the local level. This largely include the procurement of essential medicines and medical supplies to ensure the delivery of the BHCS. In this context, the procurement capacity of the local government need to be enhanced in terms of forecasting, technical evaluation, and quality assurance for the essential drugs and medical commodities procurement, as this is a critical and specialized function. In sectors like health, different evidences have shown a greater benefit by centralized or partially centralized procurement due to the larger economies of scale.^33,34^ The efficiency is also accounted to the qualification of the contracting authority. During the transition, it is quite critical that the local level is capacitated to harness the benefits of decentralization ie, increased transparency and procurement based on the local needs.

The persistent high rate of maternal and child deaths, undernutrition among children is still a challenge. Nepal Demographic and Health Survey (NDHS) 2016 shows the gains in the health sector is uneven with respect to province and wealth quintiles.^3^ Nepal needs to continue to make changes to achieve the UHC and the ambitious target of Sustainable Development Goals (SDGs).

## Opportunities and Way Forward

Despite the challenges, federalism presents ample prospects for health sector reform. Due to the proximity of the local government to the people, the federal context provides fertile ground for more effective budgeting and needs-based and evidence-based planning. Federalism also presents opportunities for increasing financial resources for health from provincial and local government. The local and provincial government, if capacitated, can prioritize and plan for the health sector from different resources like equalization grant and the local funds apart from the conditional budget from the MoHP.

It is imperative for the federal government to finalize the BHCS package and to develop necessary legislation for effective delivery. Moreover, at present there are number of fragmented health policies thus, it would be useful to form an umbrella health policy to create a streamlined service. An umbrella health policy would standardise health reform and also allow provincial and local governments to craft their policies compatible with their needs. The Public Health Act and the Reproductive Health Right Act (2018) has been approved recently. The process of developing regulations of The Public Health Act (2018), Health Insurance Act (2018), and the Safe Motherhood and Reproductive Health Right Act (2018) need to expedite for effective implementation of federal health system in spirit of constitution of Nepal. The provincial and local government will also need assistance and technical support to restructure and implement legal provisions.

Capacity development mapping and policy for the development of capacity per the changed structure and function including an institutional arrangement is needed. Ethiopia, for example, still struggles with capacity building. Nepal needs to effectively implement the Civil Service Act, 2018^36^ and regulation which are in the process of development. Local elected governments are presented with the opportunity for better health sector accountability and transparency. A multi-stakeholder forum at all three levels can advocate for the service access and quality and allow to locally mange the grievances.

In the fiscal year 2018/2019, the budget has been transferred to both province and local government. Despite the transfer of budget, there is need for the transition management plan. The set of health functions need to be delivered through new structures. Country experiences from Spain and South Africa, two of the countries which went to major political changes mainly due to federalization in the last two decades; show that transitioning to new system is a resource and time intensive process.^35^ A series of transition plans were implemented in different phases in these countries. As Nepal considers a framework for transition, a transition plan on health sector management is needed until the policy and legislative framework is fully in place.

## Conclusion

Federalism is an important opportunity for Nepal to achieve UHC. Enacting it in the health sector must be backed by legislation and quality standards, along with sound financing, logistics, human resources, and an emphasis on empowering and capacitating local and provincial governments through strengthening leadership and governance mechanisms. This context, federalism and resulting increases in healthcare accessibility and financing options present a strong prospect to strengthen the health system in Nepal.

## Acknowledgements

We would like to express our sincere thanks to all the members of the Technical Working Group and Federalism Implementation Unit under the Ministry of Health and Population (MoHP). We would like to acknowledge the support of Ms. M. Sophia Newman for her inputs during the development of this paper. Also thanks to Ms. Sophie Delamothe for her support.

## Ethical issues

Not applicable.

## Competing interests

Authors declare that they have no competing interests.

## Authors’ contributions

RT, KB, and PT conceptualized the paper, conducted literature review and wrote the first draft. TKS and SD critically reviewed and revised the draft. All authors agree on the final version of the manuscript.

## Authors’ affiliations

^1^Deutsche Gesellschaft für Internationale Zusammenarbeit (GIZ) GmbH, Kathmandu, Nepal. ^2^FHI 360 Nepal, LINKAGES Nepal Project, Kathmandu, Nepal. ^3^Province Health Directorate, Ministry of Social Development, Dhankuta, Nepal.
